# Supplementary material for: Stress-induced protein disaggregation in the endoplasmic reticulum catalysed by BiP
Source: Nat Commun. 2022 May 6;13:2501. doi: 10.1038/s41467-022-30238-2 (PMC9076838; doi:10.1038/s41467-022-30238-2)
Supplement: Supplementary file 1 — Supplementary Information [file 41467_2022_30238_MOESM1_ESM.pdf]

Supplementary Information for Stress-induced protein disaggregation in the Endoplasmic Reticulum catalysed by BiP

Eduardo Pinho Melo[1, 2*], Tasuku Konno[1], Ilaria Farace[1], Mosab Ali Awadelkareem[1], Lise R. Skov[1], Fernando Teodoro[2], Teresa P. Sancho[2], Adrienne W. Paton[3], James C. Paton[3], Matthew Fares[4], Pedro M. R. Paulo[5], Xin Zhang[4] & Edward Avezov[1 *]

[1] UK Dementia Research Institute at University of Cambridge, Department of Clinical Neurosciences, Cambridge CB2 0AH, United Kingdom

[2] CCMAR-Centro de Ciências do Mar, Universidade do Algarve, Campus de Gambelas, 8005-139 Faro, Portugal

[3] Research Centre for Infectious Diseases, Department of Molecular and Biomedical Science, University of Adelaide, Adelaide, SA, 5005, Australia

[4] Department of Chemistry, The Pennsylvania State University, University Park, Pennsylvania

[5] Centro de Química Estrutural, Instituto Superior Técnico, Universidade de Lisboa, Av. Rovisco Pais, 1049-001 Lisboa, Portugal

Supplementary Figures 1-13 and Supplementary Table 1

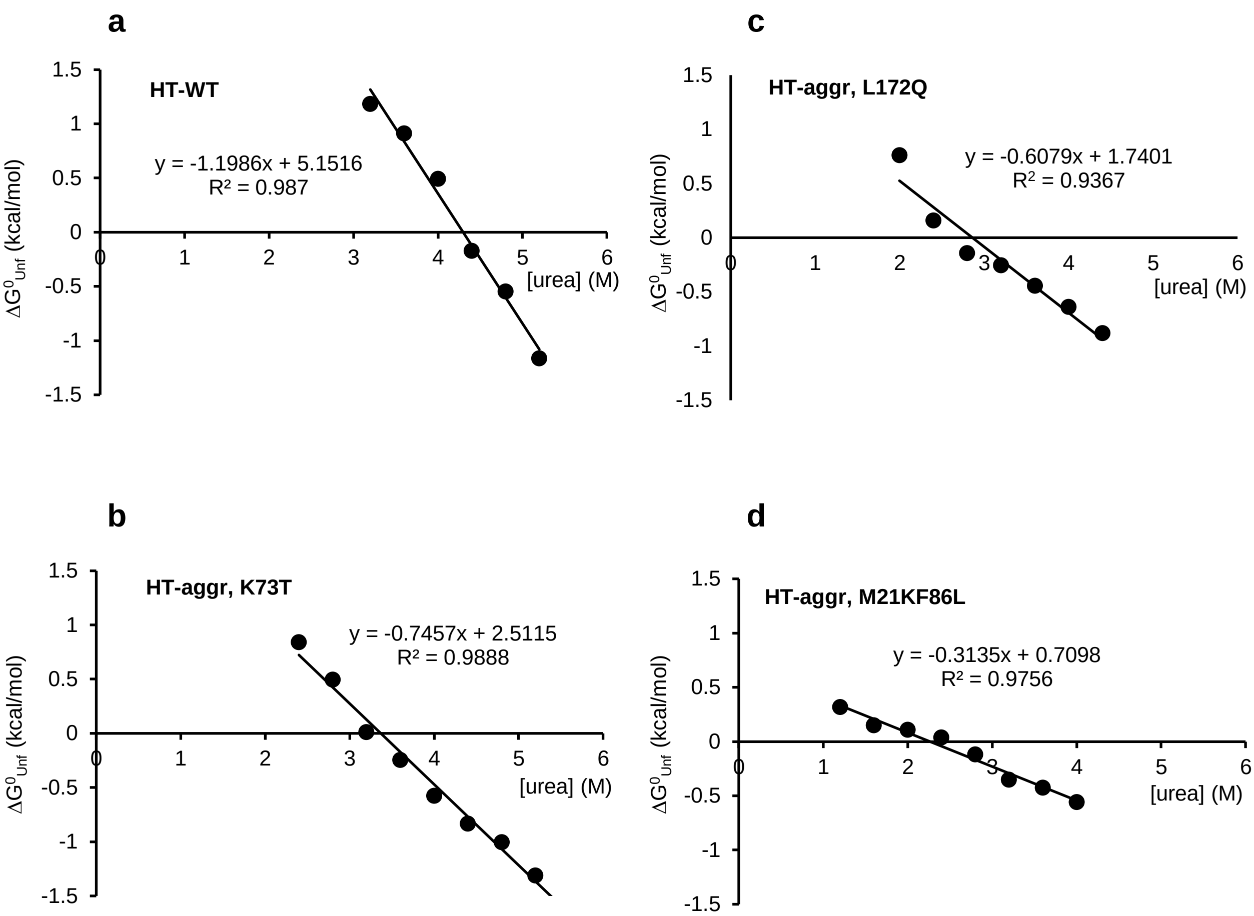

Supplementary Figure 1. Metastability measurements of the Halotag-based aggregation probes. HT-WT **(a)** or HT-aggr variants K73T **(b)**, L172Q **(c)** and M21KF86L **(d)** (30 µM)) were denatured in varying urea concentrations, and the extent of denaturation was measured through tryptophan fluorescence emission. A two-state model where only the native and the unfolded state are populated was assumed, and the Gibbs free energy of unfolding (ΔG^0_{Unf}=-RT ln K_{Unf}) at different urea concentrations within the transition region was plotted against urea concentration to determine the thermodynamic stability of HT in water at 25 ºC ($\Delta G^0_{urea} = \Delta G^0_{water}$ – *m* [urea]).

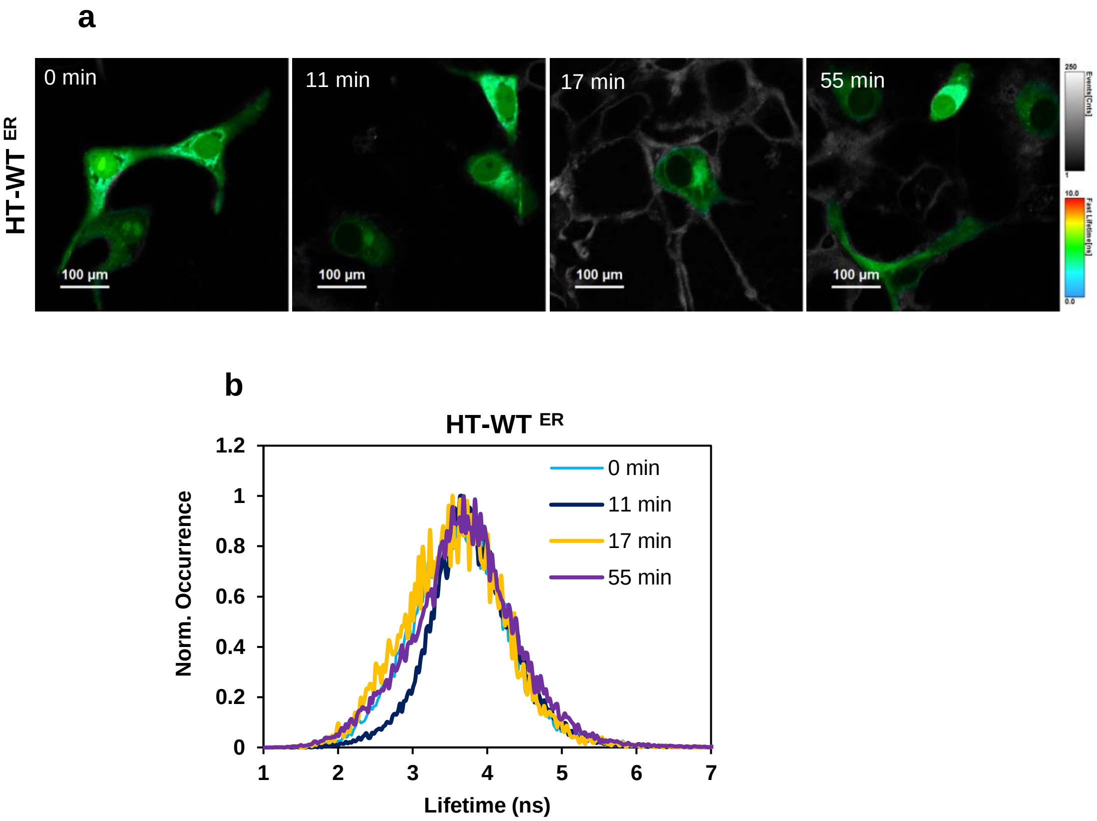

Supplementary Figure 2. Heat-shock of HT-WT^{ER}. A time-series of FLIM images, representative images from two experiments **(a)** and lifetime histograms **(b)** of HT-WT transiently expressed in the ER of COS7 cells upon heat shock treatment. Only transfected cells are lifetime coloured, gray cells for untransfected cells.

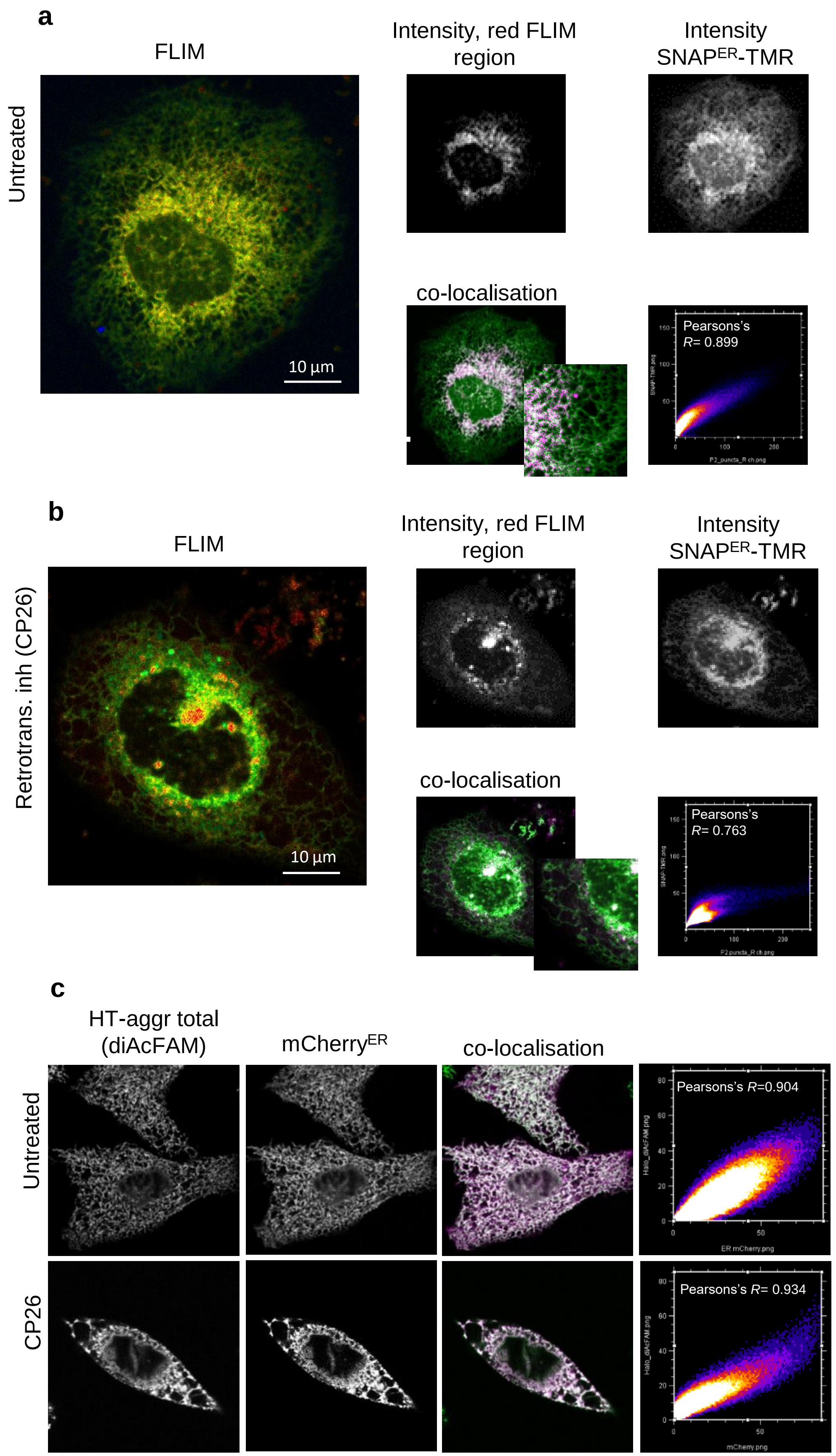

Supplementary Figure 3. Preservation of HT-aggr^{ER} localisation. (a) Co-localisation analysis of the aggregated HT-aggr^{ER} fraction (red signal extracted from the FLIM image) and an ER luminal marker (TMR-stained SNAP^{ER}, targeted to the ER with signal peptide and C-terminal KDEL, the ER-retention signal). Scatter plot correlating the fluorescence intensities of punctuated HT-aggr (X-axis) and SNAP^{ER} (Y-axis). **(b)** Co-localisation analysis as in (a) in the presence of a retro-translocation inhibitor CP26 (4.45 µM, 18 hours). **(c)** Co-localisation analysis as in (a) and (b) subjecting fluorescence intensities between the entire HT-aggr population (diAcFAM-stained) and an ER luminal marker (mCherry^{ER}). Values in insets represent Pearson's correlation coefficient (*R*). Scale bar, 10µm. Representative images from two co-localisation experiments.

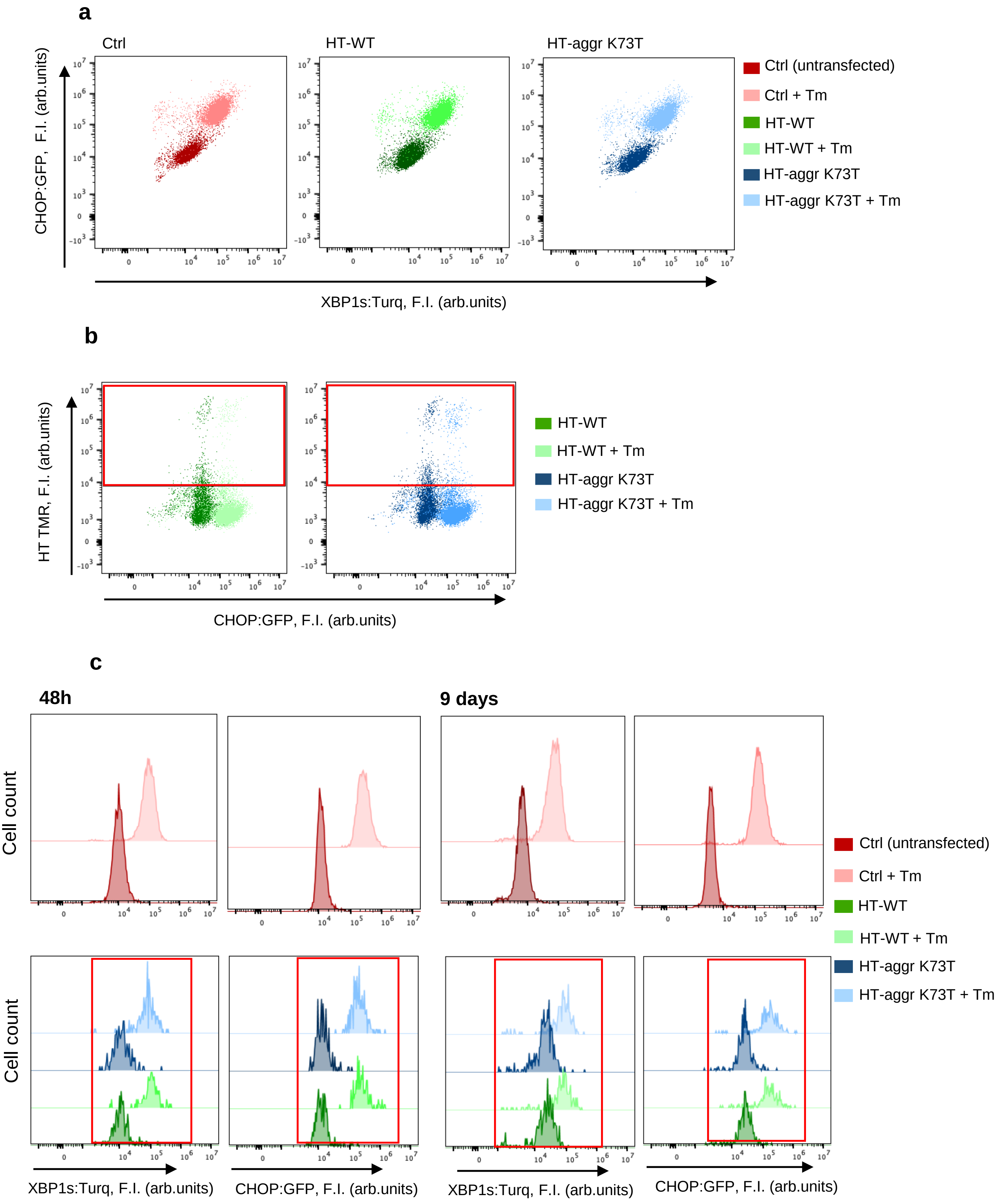

Supplementary Figure 4. Flow Cytometry analysis of UPR upon protein aggregation in the ER. (a) Scatter plots of green/blue fluorescence of S21 CHO cells expressing the UPR fluorescent reporters GFP-CHOP1 and m-Turquoise-XBP1, reflecting PERK and IRE1 pathways activation, respectively[31]. Untransfected (control, Ctrl), transfected with HT-WT^{ER} or HT-aggr^{ER}, untreated or exposed to tunicamycin (0.5 µg/mL, 6 h). **(b)** Measurements of GFP-CHOP1 UPR reporter as in (a) with detection of red fluorescence emanating from TMR Halo Ligand, identifying cells transfected with HT-WT^{ER} or HT-aggr^{ER} (red quadrants). **(c)** Histograms of fluorescence signals from GFP-CHOP1 and m-Turquoise-XBP1 in untransfected cells and HT-WT^{ER} or HT-aggr^{ER} expressing TMR-positive (red quadrants in b) cells measured following expression periods corresponding to the beginning and peak of aggregates accumulation in the ER (Fig. 2a), as indicated.

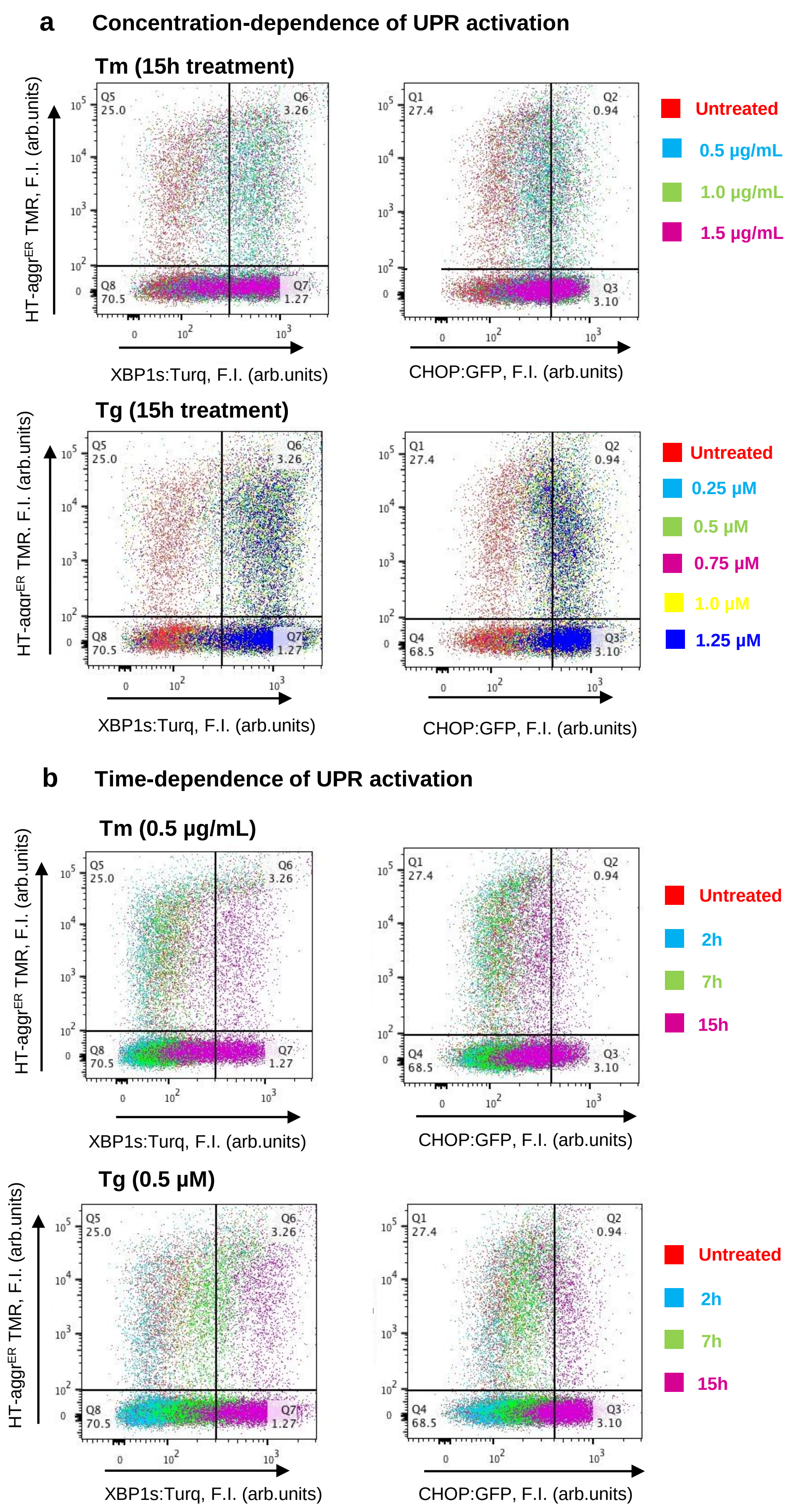

Supplementary Figure 5. Tunicamycin (Tm) and thapsigargin (Tg) concentration- and time-dependence of UPR activation. Scatter plots of green/blue fluorescence of S21 CHO cells expressing the UPR fluorescent reporters GFP-CHOP1 and m-Turquoise-XBP1, reflecting PERK and IRE1 pathways, respectively[31], transfected with HT-aggr^{ER} and labelled with TMR HT ligand. **(a)** Cells untreated or treated for 15 h with different concentration of Tm (0.5-1.5 µg/mL) or Tg (0.25-1.25 µM). **(b)** Cells untreated or treated for different times (2-15 h) with 0.5 µg/mL of Tm or 0.5 µM of Tg.

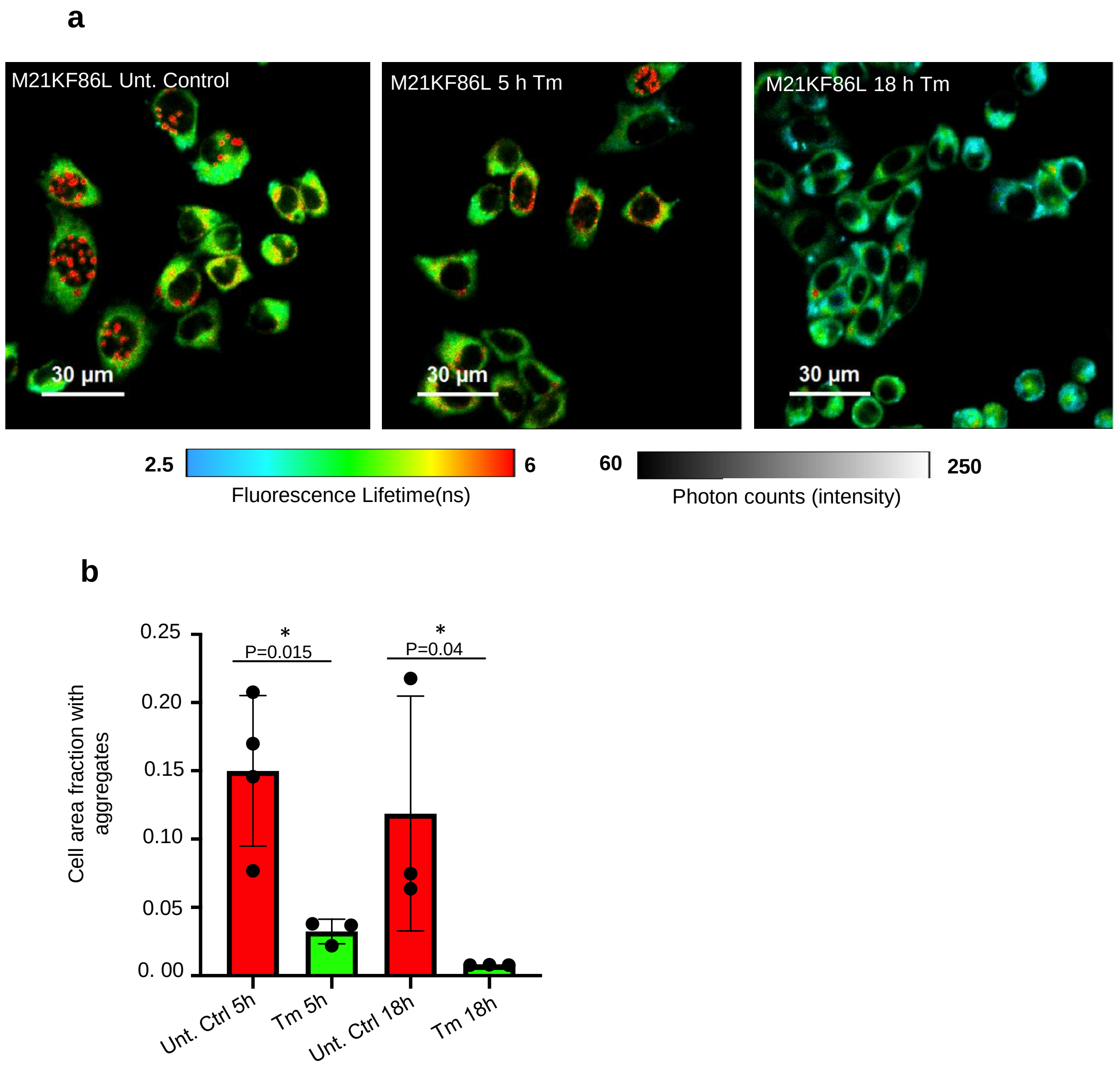

Supplementary Figure 6. Aggregates from the highly aggregation-prone HT-aggr^{ER} M21KF86L variant cleared under ER stress. (a) FLIM images of live CHO-K1 cells stably expressing HT-aggr^{ER} M21KF86L variant untreated and treated with Tm (0.5 µg/mL) for 5 h and 18h, representative images from two experiments. **(b)** Plot shows the quantification of the cell area fraction covered with aggregates. Data points (n=3 for >45 cells) equals nº of independent FLIM images, each containing multiple cells. Bar plot in panel b is presented as mean values +/- SEM. *P<0.05, unpaired T-test (two-tailed).

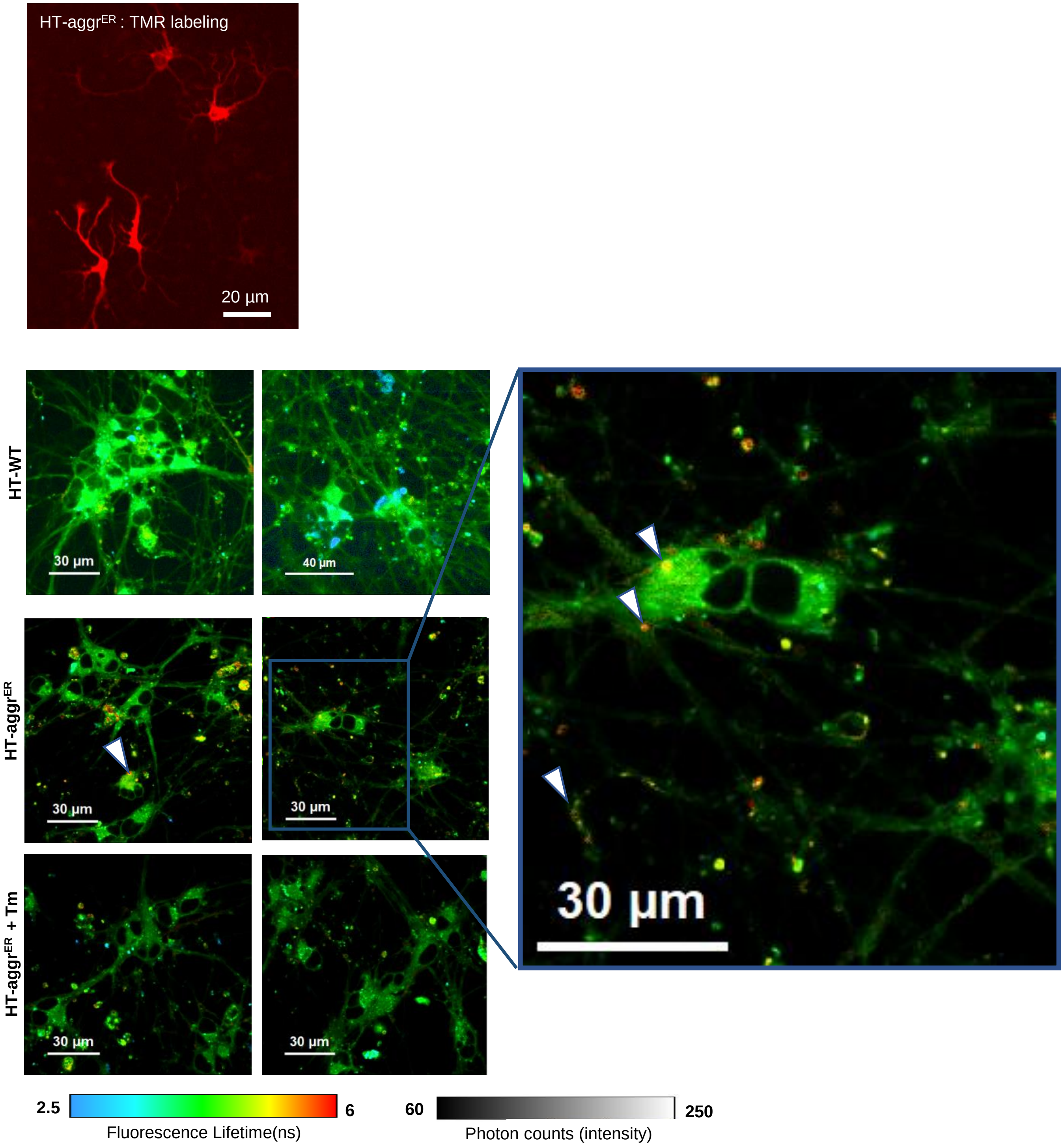

Supplementary Figure 7. ER stress-induced disaggregation of HT-aggr^{ER} in neurones. FLIM micrographs of Induced Pluripotent Stem (iPS) cells-derived cortical neuronal monolayer stably expressing HT-aggr^{ER} labelled with TMR Halo Ligand or P1 fluorophore, untreated or treated with tunicamycin, representative images from two experiments. Note the aggregation of the probe in longer lifetime regions (yellow, red, examples denoted by arrowheads).

a

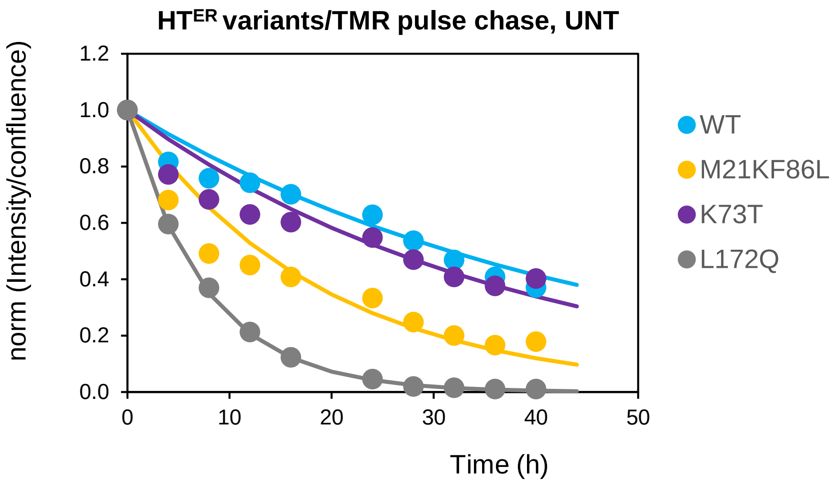

b

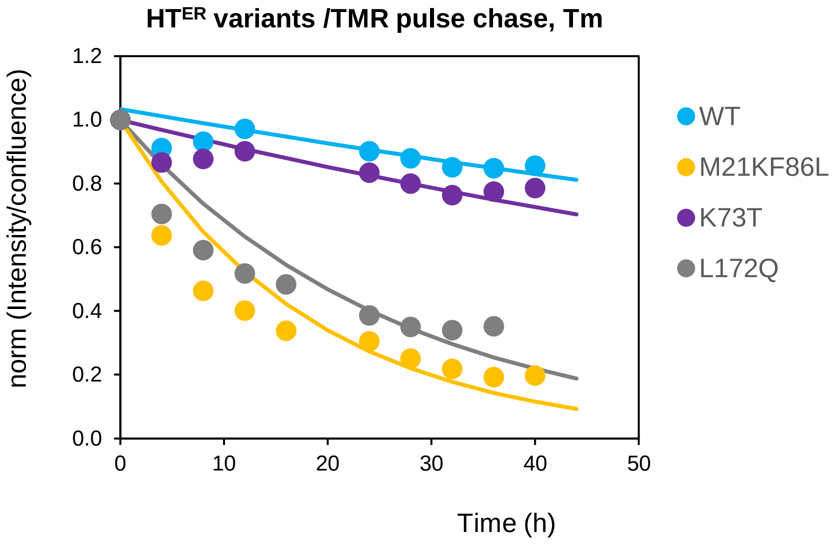

Supplementary Figure 8. Turnover rates of HT^{ER} variants. Source data for Table 1 (see Supplementary Movies 2-5). Degradation rates of stably expressed HT variants after a pulse labelling with TMR in live CHO-K1 cells in the absence **(a)** or presence of tunicamycin (0.5 µg/mL) **(b)**. The time-dependent decay of TMR fluorescence (reflecting the amount of HTs) was fitted to an exponential decay using the equation (TMR Int. Intens.$_{t}$/conf$_{t}$) = (TMR Int. Intens.$_{t0}$ /conf$_{t0}$) exp $^{(-kd\ t)}$ where TMR Int. Intens. is the TMR integrated fluorescence intensity, conf is cell confluence, and k_d is the HT degradation rate constant.

a

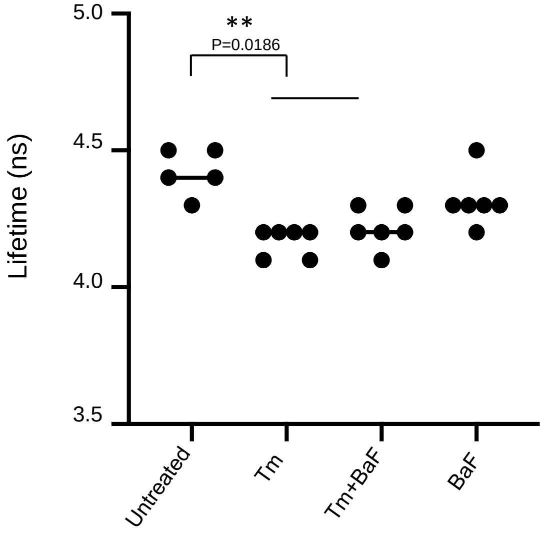

b

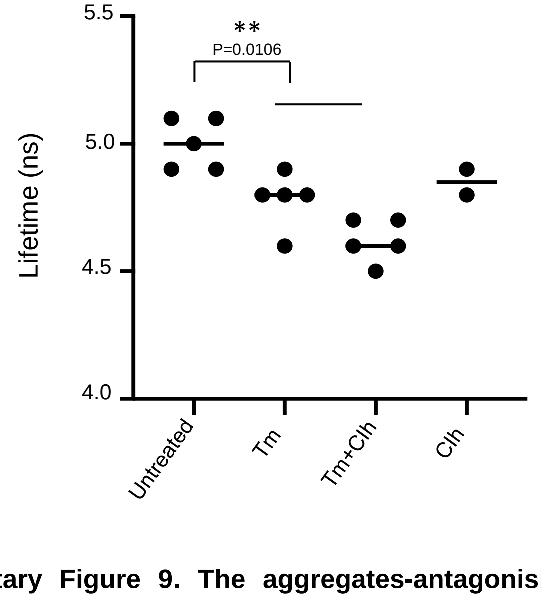

Supplementary Figure 9. The aggregates-antagonising effect of the ER stressors is insensitive to autophagosomal/lysosomal blockers. (a) Plots of mean +/- SD of fluorescence lifetime values measured as in Fig. 2b, of cells treated with Tm (0.5 µg/ml) in the presence or absence of the autophagy inhibitor Bafilomacyn (100 nM) or **(b)** the lysosomal inhibitor Chloroquine (10 µM). Data points n in panels a and b equals nº of independent FLIM images, each containing multiple cells (n=6 for >290 cells and n=5 for >200 cells in panels a and b, respectively). **P<0.01, ****P<0.0001, unpaired T-test (two-tailed).

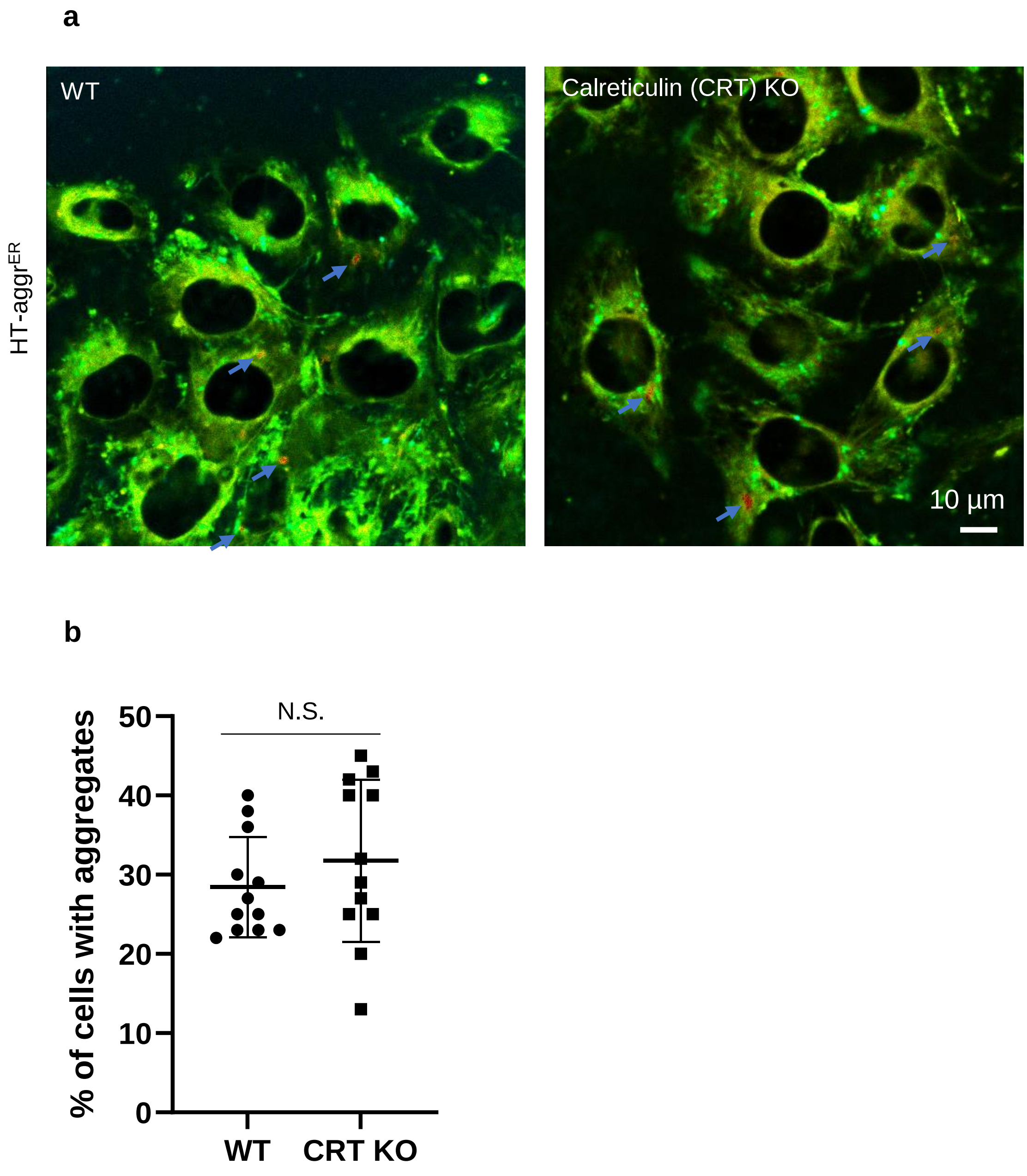

Supplementary Figure 10. Calreticulin-unrelated accumulation of HT-aggregate. (a) Representative FLIM images from two independent experiments, of HT-aggr^{ER}-stably expressing wildtype (WT) and Calreticulin knockout (CRT KO) mouse embryonic fibroblasts. **(b)** Plot shows the percentage of cells with FLIM-detectable aggregates (apparent as red puncta, exemplified by blue arrows). Data points n in panels b equals nº of independent FLIM images, each containing multiple cells (n=12 for >143 cells). N.S., not significant, unpaired t-test (two-tailed)

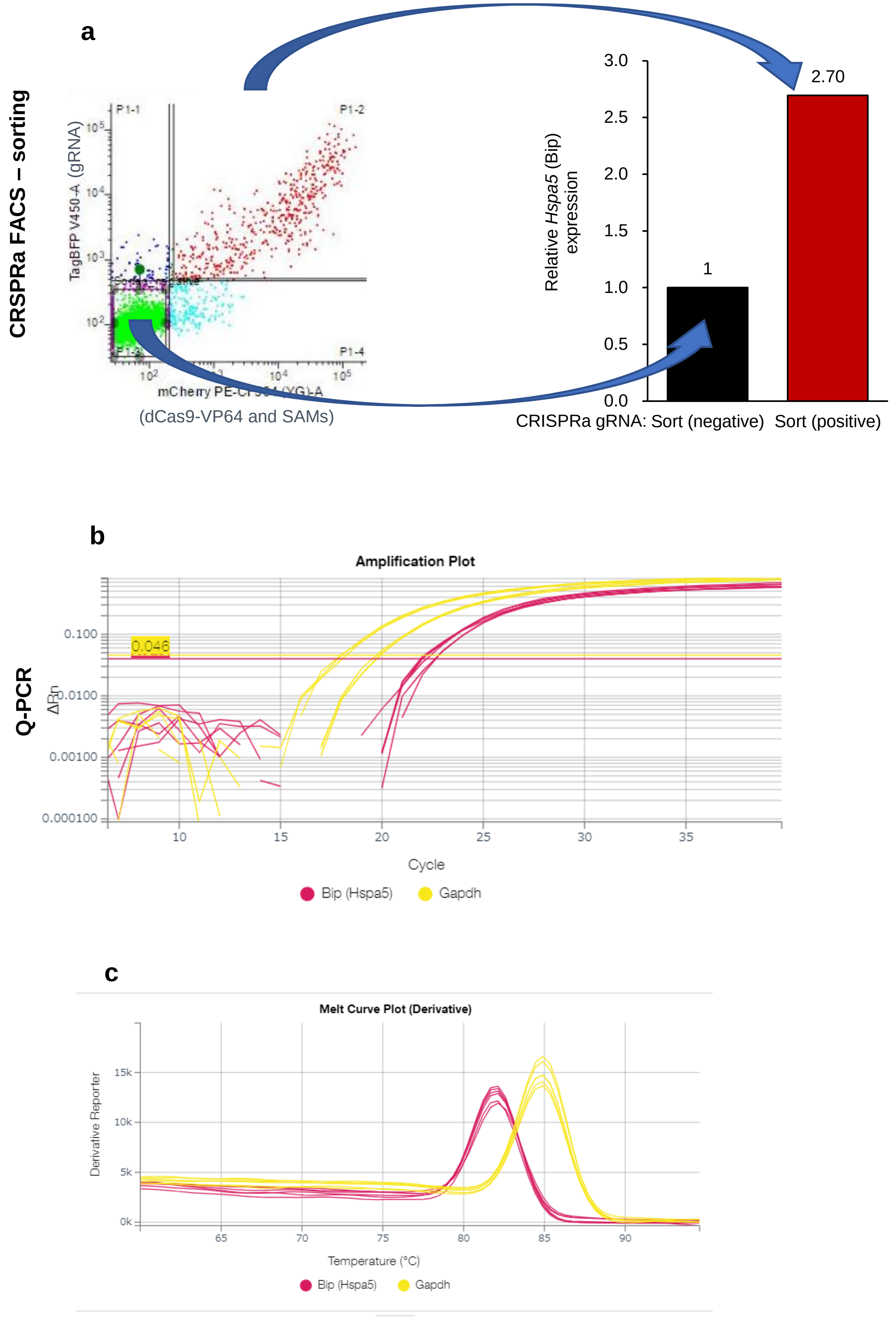

Supplementary Figure 11. Validation of CRISPRa targeting BiP (Hspa5). (a) CHO-K1 cells were transfected with gRNA and dCas9-VP64/SAM expression vectors (indicated by TagBFP and mCherry, respectively). 24 hours post-transfection, CRISPRa-occurring cells were sorted from both TagBFP- and mCherry-positive population together with the negative population as control. RNA extraction and qRT-PCR were performed as described in materials and methods. Relative gene expression of BiP (Hspa5) was calculated using the delta-delta Ct method. Amplification plot **(b)** and Melting curve plots **(c)** point to the cycle number beyond the threshold and the absence of non-specific amplification, respectively.

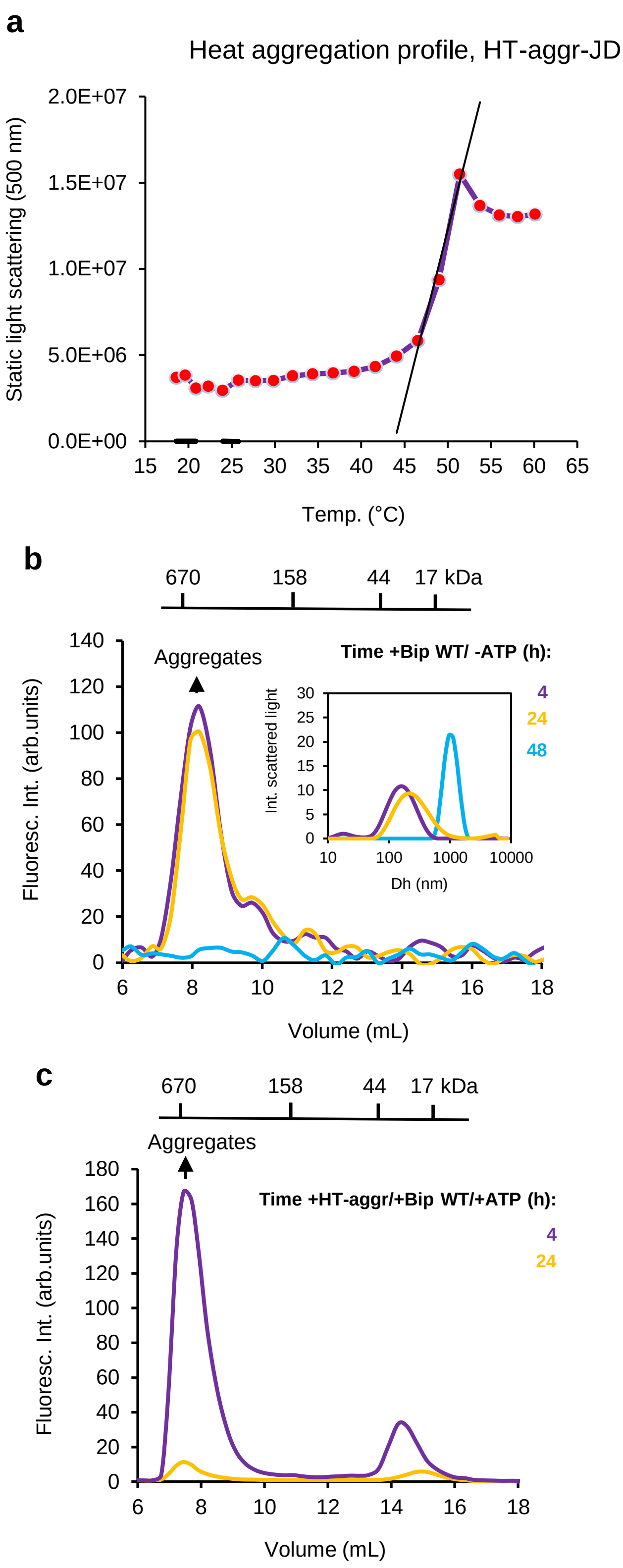

Supplementary Figure 12. Aggregation of HT-aggr-JD and HT-aggr in vitro. (a) Thermal induced aggregation of HT-aggr (K73T) fused to the J domain (HT-aggr-JD) measured by static light scattering at 500 nm to define the range of temperature where protein aggregation occurs. The onset of aggregation occurred at 43.4 ± 1.2°C as calculated from the linear extrapolation of the increase in static light scattering. Note, at 53°C HT-aggr is entirely aggregated (confirmed by gel filtration chromatography, see Fig. 5b. **(b)** Fluorescence traces of chromatograms for the P2-labelled HT-aggr-JD (pre-aggregated) in the presence of BiP but absence of ATP for the same time course as in Fig. 5d-f. Dynamic light scattering measurements showing the hydrodynamic diameter (Dh) of P2-labelled HT-aggr-JD (pre-aggregated) for the same time course were plotted in the inset to unveil the growth of the aggregates in the absence of ATP and explain why aggregates are retained in the pre-filter of the column leading to a decreased amount eluted over time. **(c)** Fluorescence traces of chromatograms for the P2-labelled HT-aggr (no J domain, pre-aggregated) in the presence of BiP and ATP for 24h to show that the aggregated probe lacking JD continue growing to a point where they are too large to pass the chromatography filter.

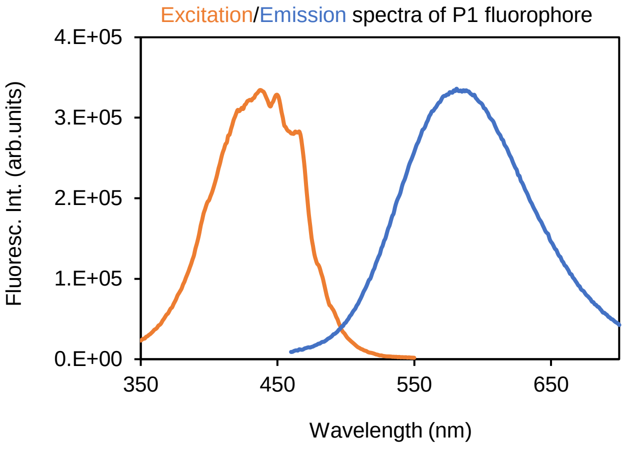

Supplementary Figure 13. Steady-state fluorescence of P1 HT fluorophore. Excitation (emission at 580 nm) and emission (excitation at 450 nm) spectra of P1 HT fluorophore in PBS.

Supplementary Table 1. List of plasmids.

Plasmid name	Expression host	Encoded protein	Origin
SP_H6_HaloK73T_KDEL_pCMV1	Mammalian ER	HT-K73T	This Study* (Addgene #183686)
pFLAG_HaloK73T_cyto	Mammalian cytosol	HT-K73T	This Study* (Addgene #183687)
SP_H6_Halo_WT_KDEL_pBABEpu	Mammalian ER	HT-WT	This Study* (Addgene #183688)
SP_H6_Halo_K73T_KDEL_pBABEpu	Mammalian ER	HT-K73T	This Study* (Addgene #183689)
SP_H6_Halo_L172Q_KDEL_pBABEpu	Mammalian ER	HT-L172Q	This Study* (Addgene #183690)
SP_H6_Halo_M21K_F86L_KDEL_pBABEpu	Mammalian ER	HT-M21KF86L	This Study* (Addgene #183691)
pLVS-VG	Mammalian	Retroviral packaging	-
pJK3	Mammalian	Retroviral packaging	-
pCMV_TATTAT_HIV	Mammalian	Retroviral packaging	-
pET30_Halotag_K73T_v2	Bacterial	HT-K73T	This study (Addgene #183692)
pET30_Halotag_K73T_Jdomain	Bacterial	HT-K73T_Jdomain	This study (Addgene #183693)
haBiP_27-654_pQE10	Bacterial	BiP-WT	PMID: 22869598
haBiP_27-654_V461F_pQE10	Bacterial	BiP-V461F	PMID: 22869598
pCRISPRa_gRNA vector	Mammalian	dCas9-VP64, SAMs	PMID: 30905739
pCRISPRa_CHO-Bip_gRNA1	Mammalian	gRNA for Bip (Hspa5)	This Study (Addgene #183693)
pCas9_VP64	Mammalian	dCas9-VP64, SAMs	PMID: 30905739
pCRISPRa_all-in-one	Mammalian	dCas9-VP64, SAMs	This Study (Addgene #183695)
pCRISPRa_all-in-one_Bip gRNA1	Mammalian	dCas9-VP64, SAMs, gRNA for Bip (Hspa5)	This Study (Addgene #183696)

* ER targeted variants made based on Halotag-ER from PMID: 30224760, K73T mutation identified in PMID: 28557281.
