# Supplementary material for: Stress-induced protein disaggregation in the endoplasmic reticulum catalysed by BiP
Source: Nat Commun. 2022 May 6;13:2501. doi: 10.1038/s41467-022-30238-2 (PMC9076838; doi:10.1038/s41467-022-30238-2)
Supplement: Supplementary file 3 — Description of Additional Supplementary Files [file 41467_2022_30238_MOESM3_ESM.pdf]

Description of Additional Supplementary Files

File name: Supplementary Movie 1

Description: **3D FLIM image of HT-aggr(ER).** High resolution fast 3D FLIM image of CHO-K1 cells to show the intra-ER localisation of HT-aggr protein aggregates.

File name: Supplementary Movie 2

Description: **Turnover of HT-aggr(ER).** Turnover rates of HT-aggr(ER) (K73T) labelled with HaloTag ligand TMR used to calculate HT-aggr degradation rate constant and half-life time.

File name: Supplementary Movie 3

Description: **Turnover of HT WT(ER).** Turnover rates of HT WT(ER) labelled with HaloTag ligand TMR used to calculate HT WT degradation rate constant and half-life time.

File name: Supplementary Movie 4

Description: **Turnover of HT-aggr(ER) in the presence of tunicamycin.** Turnover rates of HT-aggr(ER) (K73T) labelled with HaloTag ligand TMR in the presence of tunicamycin (0.5 µg/mL) used to calculate HT-aggr degradation rate constant and half-life time.

File name: Supplementary Movie 5

Description: **Turnover of HT WT(ER) in the presence of tunicamycin.** Turnover rates of HT WT(ER) (K73T) labelled with HaloTag ligand TMR in the presence of tunicamycin (0.5 µg/mL) used to calculate HT WT degradation rate constant and half-life time.
