# Supplementary material for: Stress-induced protein disaggregation in the endoplasmic reticulum catalysed by BiP
Source: Nat Commun. 2022 May 6;13:2501. doi: 10.1038/s41467-022-30238-2 (PMC9076838; doi:10.1038/s41467-022-30238-2)
Supplement: Supplementary file 9 — Reporting Summary [file 41467_2022_30238_MOESM9_ESM.pdf]

nature research

Corresponding author(s): Eduardo Pinho Melo

Last updated by author(s): 2022/03/14

# Reporting Summary

Nature Research wishes to improve the reproducibility of the work that we publish. This form provides structure for consistency and transparency in reporting. For further information on Nature Research policies, see our Editorial Policies and the Editorial Policy Checklist.

## Statistics

For all statistical analyses, confirm that the following items are present in the figure legend, table legend, main text, or Methods section.

| n/a | Confirmed | |
|---|---|---|
| ☐ | ☒ | The exact sample size (*n*) for each experimental group/condition, given as a discrete number and unit of measurement |
| ☒ | ☐ | A statement on whether measurements were taken from distinct samples or whether the same sample was measured repeatedly |
| ☐ | ☒ | The statistical test(s) used AND whether they are one- or two-sided<br>*Only common tests should be described solely by name; describe more complex techniques in the Methods section.* |
| ☒ | ☐ | A description of all covariates tested |
| ☒ | ☐ | A description of any assumptions or corrections, such as tests of normality and adjustment for multiple comparisons |
| ☐ | ☒ | A full description of the statistical parameters including central tendency (e.g. means) or other basic estimates (e.g. regression coefficient) AND variation (e.g. standard deviation) or associated estimates of uncertainty (e.g. confidence intervals) |
| ☐ | ☒ | For null hypothesis testing, the test statistic (e.g. *F*, *t*, *r*) with confidence intervals, effect sizes, degrees of freedom and *P* value noted<br>*Give P values as exact values whenever suitable.* |
| ☒ | ☐ | For Bayesian analysis, information on the choice of priors and Markov chain Monte Carlo settings |
| ☒ | ☐ | For hierarchical and complex designs, identification of the appropriate level for tests and full reporting of outcomes |
| ☐ | ☒ | Estimates of effect sizes (e.g. Cohen's *d*, Pearson's *r*), indicating how they were calculated |

*Our web collection on statistics for biologists contains articles on many of the points above.*

## Software and code

Policy information about availability of computer code

| | |
|---|---|
| Data collection | No software was used for data collection besides the ones from instruments manufacturers. CytExpert software, version 2.3.0.84 |
| Data analysis | Sympho Time 64 PicoQuant software (version 2.4); Icy platform (version 2.3.0.0), Sartorius Incucyte analysis software (version 2021C); FlowJo software (version 10.6.0) |

For manuscripts utilizing custom algorithms or software that are central to the research but not yet described in published literature, software must be made available to editors and reviewers. We strongly encourage code deposition in a community repository (e.g. GitHub). See the Nature Research guidelines for submitting code & software for further information.

## Data

Policy information about availability of data

All manuscripts must include a data availability statement. This statement should provide the following information, where applicable:

- Accession codes, unique identifiers, or web links for publicly available datasets
- A list of figures that have associated raw data
- A description of any restrictions on data availability

The data that support the findings of this study are available in a source data file as supplementary information and from the corresponding authors upon reasonable request. Plasmids and sequence information generated in this study is available through Addgene.

# Field-specific reporting

Please select the one below that is the best fit for your research. If you are not sure, read the appropriate sections before making your selection.

☒ Life sciences ☐ Behavioural & social sciences ☐ Ecological, evolutionary & environmental sciences

For a reference copy of the document with all sections, see nature.com/documents/nr-reporting-summary-flat.pdf

# Life sciences study design

All studies must disclose on these points even when the disclosure is negative.

| | |
|---|---|
| Sample size | Sample size, i.e., number of cells imaged for each condition was chosen to be statistically significant as detailed in the legend of figures |
| Data exclusions | No data was excluded from the analysis |
| Replication | At least three replicates were carried out successfully for each experiment and all were considered to mean and SEM |
| Randomization | Cells were randomized before being subjected to different treatments |
| Blinding | Blinding was not relevant to this study as no groups were established |

# Reporting for specific materials, systems and methods

We require information from authors about some types of materials, experimental systems and methods used in many studies. Here, indicate whether each material, system or method listed is relevant to your study. If you are not sure if a list item applies to your research, read the appropriate section before selecting a response.

## Materials & experimental systems

| n/a | Involved in the study |
|---|---|
| ☒ | ☐ Antibodies |
| ☐ | ☒ Eukaryotic cell lines |
| ☒ | ☐ Palaeontology and archaeology |
| ☒ | ☐ Animals and other organisms |
| ☒ | ☐ Human research participants |
| ☒ | ☐ Clinical data |
| ☒ | ☐ Dual use research of concern |

## Methods

| n/a | Involved in the study |
|---|---|
| ☒ | ☐ ChIP-seq |
| ☐ | ☒ Flow cytometry |
| ☒ | ☐ MRI-based neuroimaging |

## Eukaryotic cell lines

Policy information about cell lines

| | |
|---|---|
| Cell line source(s) | COS7, CHO-K1, HEK293T used in the present study were from ATCC (ATCC numbers in Materials). CHO-K1 CHOP-GFP Xbp1-Mturquoise was obtained from professor David Ron (Cambridge Institute for Medical Research, University of Cambridge). Human iPS cell line was obtained from Dr. Michael E. Ward (NIH). Mouse embryonic fibroblasts knockout for calreticulin and their wild type counterparts were obtained from Dr. Marek Michalak (University of Alberta, Alberta, Canada). |
| Authentication | Cell authentication was based on morphological characteristics by eye observation. CHO-K1 CHOP-GFP and Xbp1-MTurquoise reporters cell line was authenticated by the response to ER stressors. Human iPS cell line was authenticated by successful differentiation into the cortical neurons. |
| Mycoplasma contamination | Mycoplasma contamination was tested periodically and no contamination was detected |
| Commonly misidentified lines (See ICLAC register) | No commonly misidentified cell lines were used. |

## Flow Cytometry

### Plots

Confirm that:

☒ The axis labels state the marker and fluorochrome used (e.g. CD4-FITC).

☒ The axis scales are clearly visible. Include numbers along axes only for bottom left plot of group (a 'group' is an analysis of identical markers).

☒ All plots are contour plots with outliers or pseudocolor plots.

☒ A numerical value for number of cells or percentage (with statistics) is provided.

### Methodology

| | |
|---|---|
| Sample preparation | Cells were trypisinized, centrifuged (1000 g, 3 min), suspended into the FACS buffer (PBS containing 0.5% BSA), and filtered through the 40 µm cell strainer (BD). |
| Instrument | CytoFLEX S (Beckman-Coulter) |
| Software | Data was collected using CytExpert (Beckman-Coulter) and analyzed using FlowJo (BD). |
| Cell population abundance | Post-sort fractions were >95% as confirmed by post-sort FACS and microscope experiments. |
| Gating strategy | FSC-A/SSC-A gate was used to distinguish cells and debris. Subsequently, singlet cell population was defined using FSC-H/FSC-A and SSC-H/SSC-A gating to get rid of the high FSC-A/SSC-A populations. Halo protein-expressing cells were determined as the TMR fluorescence positive population, which was defined using the control sample (TMR-stained untransfected cells). |

☒ Tick this box to confirm that a figure exemplifying the gating strategy is provided in the Supplementary Information.
